# Who’s your coach? The relationship between coach characteristics and birth attendants’ adherence to the WHO Safe Childbirth Checklist

**DOI:** 10.12688/gatesopenres.13118.1

**Published:** 2020-07-20

**Authors:** Emily R. George, Rebecca Hawrusik, Megan Marx Delaney, Nabihah Kara, Tapan Kalita, Katherine E.A. Semrau

**Affiliations:** 1 Ariadne Labs | Brigham & Women's Hospital and Harvard TH Chan School of Public Health, Boston, MA, 02135, USA; 2 Population Services International, Lucknow, India

**Keywords:** checklists, maternal health services, quality improvement, in-service training, provider education, coaching, supportive supervision

## Abstract

**Background:** Research demonstrates that coaching is an effective method for promoting behavior change, yet little is known about which attributes of a coach make them more or less effective. This *post hoc*, sub-analysis of the BetterBirth trial used observational data to explore whether specific coaches’ and team leaders' characteristics were associated with improved adherence to essential birth practices listed on the World Health Organization Safe Childbirth Checklist.

**Methods:** A descriptive analysis was conducted on the coach characteristics from the 50 BetterBirth coaches and team leaders. Data on adherence to essential birth practices by birth attendants who received coaching were collected by independent observers. Bivariate linear regression models were constructed, accounting for clustering by site, to examine the association between coach characteristics and attendants’ adherence to practices.

**Results:** All of the coaches were female and the majority were nurses. Team leaders were comprised of both males and females; half had clinical backgrounds. There was no association between coaches’ or team leaders’ characteristics, namely gender, type of degree, or years of clinical training, and attendants’ adherence to essential birth practices. However, a significant inverse relationship was detected between the coach or team leader’s age and years of experience and the birth attendants’ adherence to the checklist.

**Conclusion: **Younger, less experienced coaches were more successful in promoting essential birth practices adherence in this population. More data is needed to fully understand the relationship between coaches and birth attendants.

## Background

Coaching is a successful strategy for changing individuals' performance and behaviors within industries such as sports ^1^, business ^2^, and health care ^3– 5^. Coaching, which differs from clinical training, focuses on strategically supporting someone as they determine how to put knowledge into practice. Among clinicians, there is often a disconnect between theoretical knowledge and practical application of clinical skills ^3^. Employing a coaching-based approach can be a critical method to support clinicians’ progress from beginner to expert in assessment and intervention decisions ^6– 8^.

Although the literature demonstrates that coaching is an effective method for promoting behavior change, little is known about which coach attributes are effective. This *post hoc*, sub-analysis of the BetterBirth trial used observational data to explore whether specific coaches’ and team leaders’ characteristics were associated with improved adherence by birth attendants to essential birth practices (EBPs) listed on the WHO Safe Childbirth Checklist (SCC) ^9^.

## Methods

The BetterBirth Trial, a large cluster-randomized controlled trial conducted in Uttar Pradesh, India, demonstrated that a coaching-based implementation of the SCC increased birth attendants’ adherence to EBPs, but had no effect on maternal/perinatal health outcomes ^9^. The original trial took place in 120 facilities across 24 districts of Uttar Pradesh ^9^. At each intervention facility (n=60) and its matched control site (n=60), patients were enrolled two months after the launch of the coaching intervention; health outcomes of women and their newborns were collected at 7 days postpartum ^9^.

Intervention sites were assigned coaches for 8 months to empower birth attendants to identify and resolve the barriers they faced while using the SCC ^4^. Team leaders attended every other visit with coaches to provide supportive supervision. In a sub-set of 30 facilities, independent observers (neither coaches nor staff) documented birth attendant’s adherence to practices. Observers recorded data on all practices within a specific time frame (from admission, just before delivery, within 1 minute of delivery, and within 1 hour of delivery); deliveries were observed for 1 or more pause points. Observation of practices was limited to practical and observable interactions between provider and patient or provider actions to ready supplies ^9^. Data collection took place 2 months and 6 months after coaching. Only independent observer data from intervention facilities (15 facilities) are included in this analysis, as control facilities did not receive coaching.

Using data collected during the main trial, we conducted a descriptive analysis on the coach characteristics and the relationship of those attributes and adherence to the behaviors on the WHO Safe Childbirth Checklist. We included information from the 50 BetterBirth coaches and team leaders; a subset of which (n=17 coaches or team leaders) provided coaching at facilities where independent observation was completed. Using data from the study hiring database, the variables on coaches/team leaders’ gender, age, professional degree, years of clinical training and years of experience were used in the analysis. The roles of coach and team leaders for the analysis were based on the individual’s initial role when the trial started as some coaches graduated to team leader roles throughout the trial. All coaches and team leaders were included in the descriptive analysis; only the coaches and team leaders in the subset of facilities with independent observer data were included in the subsequent models. STROBE reporting guidelines were used in the submission of this study ^10^.

Generalized linear models were constructed, accounting for clustering of births within site, to examine the association between coach characteristics and birth attendants’ adherence to EBPs. The models were estimated using generalized estimating equations ^11^. Separate models were created for coaches and team leaders. Practice adherence data was collected by independent observers. Practice adherence was calculated as a summary score of the 18 EBPs for each birth and each practice was weighted equally. The EBP score was then assigned to each coach who visited the facility where the observation took place, as coaches attempted to provide at least one coaching session to every birth attendant at a facility. The parameter estimates for the model can be interpreted as the difference in mean EBP scores between the different levels of demographic characteristics in the study. All statistical analyses were performed in SAS version 9.4 (SAS Institute, Cary, NC, USA).

## Results

All coaches were female and the majority were nurses. Team leaders were comprised of both males and females; half of which had clinical backgrounds ( Table 1 ^12^). The full complement of coaches and team leaders were relatively similar to the subset of coaches working at facilities where independent observations occurred. The only difference is the proportion of males in the team leader group (63%) and males in the team leader analytic dataset (29%).

**Table 1.** Characteristics of coaches and team leaders.

	Coaches (overall) N=34	Coaches (Included in the analysis) N=10	Team leaders (overall) N=16	Team leaders (Included in the analysis) n=7
**Characteristic**				
**Gender n (%)**				
Female	34 (100%)	10 (100%)	6 (37%)	5 (71%)
Male	0 (0%)	0 (0%)	10 (63%)	2 (29%)
**Professional degree n (%)**				
Physician	0 (0%)	0 (0%)	7 (44%)	3 (43%)
Nurse	31 (91%)	8 (80%)	0 (0%)	1 (14%)
Other (non-clinical)	2 (6%)	1 (10%)	9 (56%)	3 (43%)
Unknown	1 (3%)	1 (10%)	0 (0%)	0 (0%)
**Mean age (Years) (Range)**	27 (22–54)	29 (22–54)	33 (27–43)	34 (30 – 43)
**Mean years of clinical** **training (Range)**	3 (0–4)	3 (0–4)	2 (0–6)	3 (0 – 6)
**Mean years of prior** **experience (Range)**	3 (0–25)	4 (1–25)	7 (2–17)	8 (2–17)

There was no association between coach’s or team leaders’ gender, type of degree, or years of clinical training and providers’ adherence to EBPs. However, a significant inverse relationship was detected between the coach’s or team leader’s age as well as years of experience and the birth attendants’ adherence to the checklist ( Table 2 ^12^). As the coach’s age increased by 10 years, the mean summary score of the 18 EBP adherence decreased by almost one checklist item (ϐ = -0.93). Similarly, as the coach’s years of experience increased the number of EBP decreased slightly (ϐ = -0.13). Similar effects were found for team leaders.

**Table 2.** The relationship between coaches/team leaders' characteristics and birth attendant's adherence evidence-based practices (EBPs).

Coaches (n= 10) (number of observed deliveries = 1052)		
Variable name	Parameter estimate (95% CI)	P-value
Nurse (1 vs 0)	-0.93 (-2.90, 1.05)	0.36
Years of clinical training	-0.17 (-0.65, 0.31)	0.49
Coach age (10 years)	-0.92 (-1.21, -0.64)	<.0001
Years of prior experience	-0.13 (-0.16, -0.10)	<.0001
Team leaders (n=7) (number of observed deliveries = 906)		
Physician (1 vs 0)	0.53 (-0.67, 1.73)	0.39
Clinical degree (1 vs 0)	0.75 (-0.32, 1.81)	0.17
Years of clinical training	0.13 (-0.09, 0.34)	0.24
Team leader age (10 years)	-1.71 (-3.23 , -0.20)	0.03
Gender (Female vs Male)	0.51 (-0.72 , 1.74)	0.41
Years of prior experience	-0.14 (-0.27, -0.02)	0.02

## Conclusion

The inverse relationship between the coaches’ age and experience and adherence to EBPs suggests that younger, less experienced coaches were more successful in promoting practice adherence. Younger coaches may have been less directive, especially when coaching birth attendants who were older and/or more experienced. Additionally, coaches and team leaders possess various learning styles; the coach foundation training may have been absorbed differently by each.

A potential limitation is the introduction of bias by independent observers. To mitigate bias, observers completed standardized training (with six-month refreshers), which included procedures for using the observation tool in practice and definitions for analyzing EBP adherence. For practical reasons, these observations were performed at nonrandomly selected sites during daytime hours, which potentially limits generalizability to unobserved births ^4^. While the number of coaches included in the analytic dataset is small, the total number of observations of care was substantial.

The notion that coaching has positive effects on individual behavior change outcomes is well-supported in the literature. However, a paucity of studies explore the various dynamics between coach and coachee relationships suggests that more information is needed to fully understand the relationship between coaches and birth attendants ^13^. One survey of nearly 300 individuals in 34 different countries conducted by the Institute for Employment Studies found that factors such as age and gender of their coach were less important to coachees ^14^. The most important quality of a coach to a coachee was that the coach displayed acceptance of the individual ^14^. This matches our broader experience at Ariadne Labs, where we have often seen that softer skills (i.e., established relationships between the coach and the coachee, the coach’s disposition and personal style) produce higher rates of sustained behavior change, but these factors are difficult to measure. Future research should include a mixed methods approach to explore how factors like personal styles, cultural dynamics, and hierarchy affect coaching content uptake.

## Ethics compliance

At trial initiation, birth attendants and facility staff provided written consent to participate. Before an independent observer collected data, the birth attendant verbally reconfirmed agreement; laboring women who were observed provided written consent. Electronic data were deidentified and stored in a Health Insurance Portability and Accountability Act–compliant database to ensure participant privacy. In directly observed births, women or their surrogates provided written consent for observation. The study protocol was approved by the Community Empowerment Lab (CEL) Ethics Review Committee (Ref no: 2014006), Jawaharlal Nehru Medical College Ethical Review Committee (Ref no: MDC/IECHSR/2015-16/A-53), the Institutional Review Board of the Harvard T.H. Chan School of Public Health (Protocol 21975-102), the Population Services International Research Ethics Board (Protocol ID: 47.2012), and the Ethical Review Committee of the World Health Organization (Protocol ID: RPC 501), and the Indian Council of Medical Research. The protocol was reviewed and reapproved on an annual basis.

## Data availability

### Underlying data

Harvard Dataverse: Who's Your Coach? The relationship between coach characteristics and birth attendants' adherence to the WHO Safe Childbirth Checklist. https://doi.org/10.7910/DVN/BCMETW ^12^

This project contains the following underlying data:

▪ coachtl_chars.sas7bdat (SAS dataset with demographic characteristics of coach team leaders)
▪ coachtl_checklist.sas7bdat (SAS dataset with adherence to checklist behaviors in coach team leader facilities)
▪ Coach_characteristics_DataDictionary.tab (Data Dictionary for the demographic characteristics datasets for Coaches and Coach Team Leaders with variable names, type, length, format, informat and label)
▪ coach_chars.sas7bdat (SAS dataset with demographic characteristics of coaches)
▪ coach_checklist.sas7bdat (SAS dataset with adherence to checklist behaviors in coached facilities)
▪ Coach_CoachTL_Checklist_DataDictionary.tab (Data dictionary for the datasets on adherence to Checklist behaviors listed above with variable names, type, length, format, informat and label)

Data are available under the terms of the Creative Commons Zero "No rights reserved" data waiver (CC0 1.0 Public domain dedication).
