# Supplementary material for: Who’s your coach? The relationship between coach characteristics and birth attendants’ adherence to the WHO Safe Childbirth Checklist
Source: Gates Open Res. 2020 Jul 20;4:111. [Version 1] doi: 10.12688/gatesopenres.13118.1 (PMC7417619; doi:10.12688/gatesopenres.13118.1)
Supplement: Peer review history

# Peer review: Reviewer response for version 1

**DOI:** 10.21956/gatesopenres.14297.r29125

**Published:** 2020-08-10

**Authors:** Somesh Kumar

**Affiliations:** 1 Jhpiego (an affiliate of Johns Hopkins University), Baltimore, MD, USA

This is a well written paper which shines the light on an important topic: the characteristics of a successful coach. Given the potential investments of the government in hriing of Quality Mentors, the guidance provided by this paper can inform the selection process.

 I would recommend clearly describing and differentiating the role of the coach versus a Team Leader. While the role of coach is clear, the concept of team leader is not- who is the team leader; who do they lead, what is their role?

 In the abstract, authors mention the use of Bivariate analysis. But in the methods section of the main paper, multiple background characteristics have been mentioned as variables. Any reason why a multi-variate analysis was not attempted? 

 There is a speculation made in the conclusion related to effect of training of coaches on their effectiveness.Were proficiency levels of coaches tested at any stage? It would have helpful to have that analysis.

Is the work clearly and accurately presented and does it cite the current literature?

Yes

If applicable, is the statistical analysis and its interpretation appropriate?

Partly

Are all the source data underlying the results available to ensure full reproducibility?

Yes

Is the study design appropriate and is the work technically sound?

Partly

Are the conclusions drawn adequately supported by the results?

Yes

Are sufficient details of methods and analysis provided to allow replication by others?

Yes

Reviewer Expertise:

Reproductive, Maternal, Neonatal Health; Women's Cancers; Health Systems/Quality of Care

I confirm that I have read this submission and believe that I have an appropriate level of expertise to confirm that it is of an acceptable scientific standard, however I have significant reservations, as outlined above.

# Peer review: Reviewer response for version 1

**DOI:** 10.21956/gatesopenres.14297.r29124

**Published:** 2020-08-03

**Authors:** Rosemary Ogu

**Affiliations:** 1 Department of Obstetrics and Gynaecology, University of Port Harcourt, Port Harcourt, Nigeria

The Safe Childbirth Checklist is a tool recently introduced by the World Health Organization (WHO) to reduce maternal morbidity and mortality ^1^. The WHO Safe Childbirth Checklist aims to help frontline healthcare workers prevent avoidable childbirth-related mortality/morbidity as seen in sub-Saharan Africa ^2^.

 “Who’s your coach? The relationship between coach characteristics and birth attendants’ adherence to the WHO Safe Childbirth Checklist" ^3^ is a multi-authored study on the characteristics (gender, professional degree, age, years of clinical training, and years of prior experience) and effectiveness of a coach with respect to adherence to essential birth practices by birth attendants who were coached. The authors present a sub-analysis of the BetterBirth trial using observational data to explore whether specific coaches’ and team leaders' characteristics were associated with improved adherence to essential birth practices listed on the WHO Safe Childbirth Checklist. 

 The Introduction clearly demonstrates that coaching is an effective method, yet little is known about the attributes or characteristics of a coach that make them more or less effective for promoting behavior change. 

 A descriptive analysis of the coach characteristics from the 50 BetterBirth coaches and team leaders formed the manuscript basis. Independent observers collected data on adherence to essential birth practices by birth attendants who received coaching. Bivariate linear regression models were constructed to examine the association between coach characteristics and attendants’ adherence to practices. 

 The results showed no association between the Coaches gender, type of degree, or years of clinical training, and attendants’ adherence to essential birth practices. However, a significant inverse relationship was detected between the coach age and years of experience and the birth attendants’ adherence to the checklist. 

 The authors concluded that younger, less experienced coaches were more successful in promoting essential birth practices adherence in this population and more data is needed to fully understand the relationship between coaches and birth attendants. The conclusions drawn are adequately supported by the results and the limitations were discussed. The Conclusion may be re-titled Discussion and Conclusion.

 This is a well thought out study that provides information necessary to promote behavior change. The statistical analysis and interpretation are appropriate. The work is clearly presented and cites current and relevant literature. The study design is appropriate and the work is technically sound. Independent observers (neither coaches nor staff) documented the birth attendant’s adherence to practices. The details of methods and analysis have been provided to allow replication by others. The authors have adhered to the STROBE reporting guidelines and the source data underlying the results are available and archived with a digital object identifier.

Is the work clearly and accurately presented and does it cite the current literature?

Yes

If applicable, is the statistical analysis and its interpretation appropriate?

Yes

Are all the source data underlying the results available to ensure full reproducibility?

Yes

Is the study design appropriate and is the work technically sound?

Yes

Are the conclusions drawn adequately supported by the results?

Yes

Are sufficient details of methods and analysis provided to allow replication by others?

Yes

Reviewer Expertise:

Maternal Health

I confirm that I have read this submission and believe that I have an appropriate level of expertise to confirm that it is of an acceptable scientific standard.

## References

[1] WHO Safe Childbirth Checklist Implementation Guide: improving the quality of facility-based delivery for mothers and newborns. *http://www.who.int/patientsafety/implementation/checklists/childbirth-checklist_implementation-guide/en/*.2015;

[2] : Improving Maternal Health: The Safe Childbirth Checklist as a Tool for Reducing Maternal Mortality and Morbidity.2019; 10.5772/intechopen.82616 10.5772/intechopen.82616

[3] : Who's Your Coach? The relationship between coach characteristics and birth attendants' adherence to the WHO Safe Childbirth Checklist. *Harvard Dataverse*.2020; 10.7910/DVN/BCMETW PMC741761932803131

# Peer review: Reviewer response for version 1

**DOI:** 10.21956/gatesopenres.14297.r29123

**Published:** 2020-08-03

**Authors:** Eugene Tuyishime

**Affiliations:** 1 University of Rwanda, Kigali, Rwanda; 2 OhioHealth, Columbus, Ohio, USA

Thank you for giving me the opportunity to review this research article.

 The authors talked briefly in the background section about coaching, however a more detailed literature review is needed in order to justify the gap in the literature this study is trying to address.

 More details about the study design are needed. It is unclear to the reader why the subset of 10 coaches and 7 team leaders was included in the final analysis. What happened to the remaining coaches and team leaders in table 1.

 The choice of methodology should be justified. Is this the best method to determine the attributes of a good coach? Or a mixed-method would be better? The weakness of the method and lack of a validated tool to measure coaching performance should be recognized.

 In addition, the choice of factors included in the model needs explanation.

 In the discussion section, the fact of having only female coaching should be explained.

 In the conclusion section, the sentences below are speculations and should be moved to the discussion section: "Younger coaches may have been less directive, especially when coaching birth attendants who were older and/or more experienced. Additionally, coaches and team leaders possess various learning styles; the coach foundation training may have been absorbed differently by each."

Is the work clearly and accurately presented and does it cite the current literature?

Partly

If applicable, is the statistical analysis and its interpretation appropriate?

I cannot comment. A qualified statistician is required.

Are all the source data underlying the results available to ensure full reproducibility?

Yes

Is the study design appropriate and is the work technically sound?

Partly

Are the conclusions drawn adequately supported by the results?

Partly

Are sufficient details of methods and analysis provided to allow replication by others?

Yes

Reviewer Expertise:

Global health, Quality improvement, Maternal Health, Anesthesia, and Medical Education.

I confirm that I have read this submission and believe that I have an appropriate level of expertise to confirm that it is of an acceptable scientific standard, however I have significant reservations, as outlined above.
